# Multi-Method Analysis of MRI Images in Early Diagnostics of Alzheimer's Disease

**DOI:** 10.1371/journal.pone.0025446

**Published:** 2011-10-13

**Authors:** Robin Wolz, Valtteri Julkunen, Juha Koikkalainen, Eini Niskanen, Dong Ping Zhang, Daniel Rueckert, Hilkka Soininen, Jyrki Lötjönen, the Alzheimer's Disease Neuroimaging Initiative

**Affiliations:** 1 Biomedical Image Analysis Group, Department of Computing, Imperial College London, London, United Kingdom; 2 Department of Neurology, Kuopio University Hospital, Kuopio, Finland; 3 Knowledge Intensive Services, VTT Technical Research Centre of Finland, Tampere, Finland; 4 Department of Applied Physics, University of Eastern Finland, Kuopio, Finland; 5 Institute of Clinical Medicine, Neurology, University of Eastern Finland, Kuopio, Finland; University Hospital La Paz, Spain

## Abstract

The role of structural brain magnetic resonance imaging (MRI) is becoming more and more emphasized in the early diagnostics of Alzheimer's disease (AD). This study aimed to assess the improvement in classification accuracy that can be achieved by combining features from different structural MRI analysis techniques. Automatically estimated MR features used are hippocampal volume, tensor-based morphometry, cortical thickness and a novel technique based on manifold learning. Baseline MRIs acquired from all 834 subjects (231 healthy controls (HC), 238 stable mild cognitive impairment (S-MCI), 167 MCI to AD progressors (P-MCI), 198 AD) from the Alzheimer's Disease Neuroimaging Initiative (ADNI) database were used for evaluation. We compared the classification accuracy achieved with linear discriminant analysis (LDA) and support vector machines (SVM). The best results achieved with individual features are 90% sensitivity and 84% specificity (HC/AD classification), 64%/66% (S-MCI/P-MCI) and 82%/76% (HC/P-MCI) with the LDA classifier. The combination of all features improved these results to 93% sensitivity and 85% specificity (HC/AD), 67%/69% (S-MCI/P-MCI) and 86%/82% (HC/P-MCI). Compared with previously published results in the ADNI database using individual MR-based features, the presented results show that a comprehensive analysis of MRI images combining multiple features improves classification accuracy and predictive power in detecting early AD. The most stable and reliable classification was achieved when combining all available features.

## Introduction

Alzheimer's disease (AD) is the most common cause of dementia globally and one of the major healthcare issues of the future. It has been estimated that during the next four decades the prevalence of AD will quadruple from 27 to 106 million by which time 1 in 85 persons worldwide will be living with the disease [1]. Even a modest delay of one year in disease onset and progression could reduce the number of cases by 9 million [1]. Interventions are postulated to be most effective when directed at patients at the earliest stages of the disease, which underlines the importance of early diagnosis of AD [2]. Mild cognitive impairment (MCI) is a heterogeneous syndrome that increases the risk of developing AD markedly [3]. However, not all MCI subjects convert to AD and some may even return to normal cognition [4].

The search for reliable biomarkers of AD-type pathology and predictors of disease progression among MCI subjects is ongoing. AD is characterized by neurofibrillary tangles and amyloid plaques in the brain [5]. Degenerative changes in the human neurotransmitter system lead to atrophy in selected brain regions [6]. The most promising candidate biomarkers are the ones derived from structural and functional neuroimaging as well as those measured in cerebrospinal fluid (CSF) and plasma [7]. Amyloid-based measures like the CSF-peptide A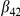 and the uptake of the PiB tracer on positron emission imaging (PET) show the earliest AD-type changes [7]. However, there is evidence that the number of amyloid plaques reach their saturation levels already by the time patients have clinically apparent symptoms of cognitive impairment [8], [9], whereas atrophy, neuronal loss, synaptic loss, and the number of tangles increase with severity of illness [10]. These findings suggest that, although amyloid-based biomarkers may be used as longitudinal markers of AD type pathology, they seem to offer only limited insight into which MCI subjects will most likely convert to AD in the near future. In a recently published dynamic model of biomarker behavior in the AD spectrum, biomarkers based on structural magnetic resonance imaging (MRI) have been shown to be correlated with a progression from MCI to AD [11]. Such biomarkers could therefore improve the accuracy of early AD diagnostics and reduce especially the amount of false positive diagnoses. Besides providing chance for a more focused and earlier intervention, structural MRI biomarkers of AD could also aid the development of new disease-modifying drugs by acting as surrogate markers of disease progression, reduce the number of subjects needed to detect significant drug effect and provide quantitative measures of treatment benefits [12].

It has been shown that the early diagnostics of AD can be improved by using multiple different biomarkers simultaneously. Usually these studies have combined MRI-based markers with biomarkers based on positron emission tomography (PET) [13], [14], cerebrospinal fluid (CSF) [15], [16] or both [17]–[19]. Achieved results vary from no additional benefit [15], [17] to significant improvement [13], [14], [16], [20]. However, availability of all three biomarkers (CSF, PET, MRI) is not very common in clinical practice since obtaining all measures is laborious for the patient and clinician, induces delays and increases the costs of the diagnosis significantly. Furthermore, measurements obtained from CSF and PET are considered invasive. Recent studies focusing on only structural MRI have reached correct classification accuracys (CCR) of 76–94% in identifying healthy controls (HC) from patients with AD and 64–82% in predicting which MCI subjects will convert to AD in the imminent future [21]–[27]. The high variation in these results can be attributed to differences in study populations as well as evaluation designs. With the Alzheimer's Disease Neuroimaging Study (ADNI) [28], a large multi-center study on MR imaging in AD has been established that is available to the wider research community. Based on a large sub-group of ADNI subjects, Cuingnet et al. [29] presented a comparison of ten MRI-based feature extraction methods and their ability to discriminate between clinically relevant subject groups. The ten methods evaluated comprise five voxel-based methods, three methods based on cortical thickness and two methods based on the hippocampus. Best sensitivity/specificity values reported are 81%/95% for AD vs HC, 70%/61% for S-MCI vs P-MCI and 73%/85% for HC vs P-MCI.

In this paper we use the ADNI database to evaluate the ability of the combination of different MR-based features to increase classification accuracy. We evaluate the power of hippocampal volume (HV), cortical thickness (CTH), tensor-based morphometry (TBM) and features extracted from a recently proposed manifold-based learning (MBL) framework to discriminate healthy controls from subjects with AD and to predict conversion from MCI to AD. For evaluation we used all 834 ADNI baseline images that were available from the ADNI webpage. Compared to previous work this paper aims at establishing the improvement in accuracy and stability that can be achieved by combining more than one MR-based feature. To the best of our knowledge it is the first comprehensive study that analyzes MRI-derived features for the full ADNI dataset. For direct comparison with the work by Cuingnet et al. [29] we also evaluated all results on the subset used in their work.

To test the influence of the classification method used, we utilized both support vector machines (SVMs) and a linear discriminant analys (LDA) to evaluate classification accuracy (CCR), sensitivity (SEN) and specificity (SPE) in each experiment.

## Materials and Methods

### Subjects

In the ADNI study, brain MR images were acquired at regular intervals after an initial baseline scan from approximately 200 cognitively normal older subjects (HC), 400 subjects with mild cognitive impairment (MCI), and 200 subjects with early AD. Detailled inclusion/exclusion criteria used for the different subject groups in ADNI are defined in [30]. The AD group has scores between 20–26 (inclusive) on the Mini-Mental State Examination (MMSE) [31], and a Clinical Dementia Rating (CDR) [32] of 0.5 or 1.0. Furthermore, these subjects fulfil the NINCDS/ADRDA criteria for probable AD [33]. MCI subjects included have MMSE scores between 24–30 (inclusive), a memory complaint, have objective memory loss measured by education adjusted scores on Wechsler Memory Scale Logical Memory II, a CDR of 0.5, absence of significant levels of impairment in other cognitive domains, essentially preserved activities of daily living, and an absence of dementia [30]. Healthy subjects have MMSE scores between 24–30 (inclusive), a CDR of 0, are non-depressed, non MCI, and nondemented. A more detailed description of the ADNI study is given in Appendix S1.

All 834 ADNI subjects (231 HC, 238 S-MCI, 167 P-MCI, 198 AD) for which a 1.5T T1-weighted MRI scan at baseline was available were included in this study. 167 subjects in the MCI group converted to AD as of July 2011. We therefore independently analysed progressive MCI (P-MCI) subjects and subjects with a stable diagnosis of MCI (S-MCI).

Table 1 shows the demographics for the 834 study subjects. Statistically significant differences in the demographics and clinical variables between the study groups were assessed using Student's unpaired t-test. In this work, the difference was considered statistically significant if p
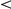
0.05 if not stated otherwise. There were more men than women in all other groups besides the AD group. MMSE scores were significantly different in the pairwise comparisons between all study groups. CDR scores of the HC and AD groups are significantly different to the ones of the two MCI groups. Healthy subjects had a significantly lower Geriatric Depression Scale (GDS) compared to all other groups. Compared to all other groups, AD subjects had significantly shorter education.

**Table 1:** Subjects.

Group	HC	S-MCI	P-MCI	AD
N	231	238	167	198
Men	52%	66%	62%	52%
Age	76.02 (5.0)	74.85 (7.8)	74.6 (7.0)	75.68 (7.7)
MMSE	29.1* (1.0)	27.3* (1.8)	26.6* (1.7)	23.3* (2.0)
CDR	0 (0)	0.49 (0.05)	0.50 (0)	0.75 (0.25)
GDS	0.83* (1.14)	1.60 (1.42)	1.53 (1.30)	1.67 (1.42)
Education	16.0 (2.8)	15.6 (3.1)	15.7 (2.9)	14.7* (3.1)
APOE4 status ( 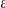 3 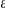 4/ 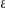 4 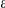 4)	23%/2%	31%/8%	50%/16%	42%/18%
Months to conversion			18.2 (10.1)	

*means statistically significant different from all other groups.

### MRI Acquisition

Standard 1.5T screening/baseline T1-weighted images obtained using volumetric 3D MPRAGE protocol with resolutions ranging from 0.9 mm
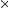
0.9 mm
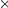
1.20 mm to 1.3 mm
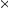
1.3 mm
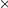
1.20 mm were included from the ADNI database. For detailed information of the MRI protocols and preprocessing steps see [34].

### Feature extraction

All fully automated feature extraction methods described below were applied to images that were preprocessed by the ADNI pipeline.

#### Hippocampal volume

Baseline hippocampal volumes were measured using an approach based on fast and robust multi-atlas segmentation [35], [36]. In this approach, multi-atlas label propagation is applied in combination with atlas selection to obtain the hippocampus segmentation. A set of hippocampus atlases is selected from a pool of atlas images according to image similarity with the query image. After registering all atlases to the query image, a spatial prior is generated from the multiple label maps. This spatial prior is then used to obtain a final segmentation based on an expectation maximization (EM) segmentation algorithm [37].

#### Cortical thickness

CTH is measured in the baseline T1-weighted structural MR images by using an automated computational surface-based method developed at the McConnell Brain Imaging Centre, Montreal Neurological Institute, McGill University, Montreal, Canada (http://www2.bic.mni.mcgill.ca/) [38]. Individual MRI volumes were registered to standard space using the ICBM152 template [39]. Intensity non-uniformities were corrected [40] before the final brain mask was calculated [41]. Tissues were segmented into white matter (WM), grey matter (GM) and cerebrospinal fluid (CSF) using the INSECT-algorithm [42] and the magnitude of PVE was estimated by using the trimmed minimum covariance determinant (TMCD) method [43]. The brains were divided automatically into two separate hemispheres and the inner and outer surfaces of the cortex were extracted according to intersections between WM and GM (white matter surface, WMS) as well as GM and CSF (grey matter surface, GMS) using the Constrained Laplacian-Based Automated Segmentation with Proximities (CLASP) algorithm [44]. The inner surface was first formed by deforming an ellipsoid polygon mesh to the shape of the WMS. GMS was obtained by further expanding the inner surface. Each polygon mesh surface consisted of 81,920 polygons and 40,962 nodes per hemisphere. The thickness of the cortex was defined at each linked node as the distance between the two concentrically linked polygon meshes on the WMS and the GMS. This t-link metric has been proven to be the simplest yet most precise way to determine cortical thickness [38]. Although MR images were transformed to standard space to allow for group analysis, thickness calculations were performed in each subject's native space. Finally, cortical thickness maps were smoothed with a 20 mm FWHM diffusion smoothing kernel to improve the signal-to-noise ratio and statistical power [45]. The described toolbox did not achieve satisfactory results on some study subjects because of i) failure in tissue segmentation and brain masking (48 subjects) and ii) failure in partial volume effect estimation (59 subjects). As a result the pipeline crashed and CTH measures were not obtained for 76 subjects (24 control, 35 MCI, 17 AD). Also the cortical model of 31 subjects (10 control, 13 MCI, 8 AD) was completely deformed and thus unusable. For these 107 subjects the CTH features were considered as missing values. CTH features used in the classification experiments are introduced below.

#### Tensor-based morphometry

The TBM analysis was performed using a multi-template approach [46], [47]. In TBM, a template image is non-rigidly registered to a study image, and, typically, the determinant of the Jacobian matrix (‘the Jacobian’) of the deformation is used to measure the voxel-level morphometry. Instead of using just one template image, we used 30 randomly selected images (10 controls, 10 MCIs, and 10 ADs) from the ADNI database as template images. The template images were used also in the classification analysis to maximize the number of subjects. Each template image was registered to a study image, and Jacobian maps were computed for each template image. To combine the results of multiple templates, all template images were registered to the mean anatomical template generated from the 30 images, and all the results were normalized to this reference space [47]. The combination of the results was performed by averaging the ROI-wise feature values of all the templates as described in detail below.

#### Manifold-based learning

**Figure 1:**
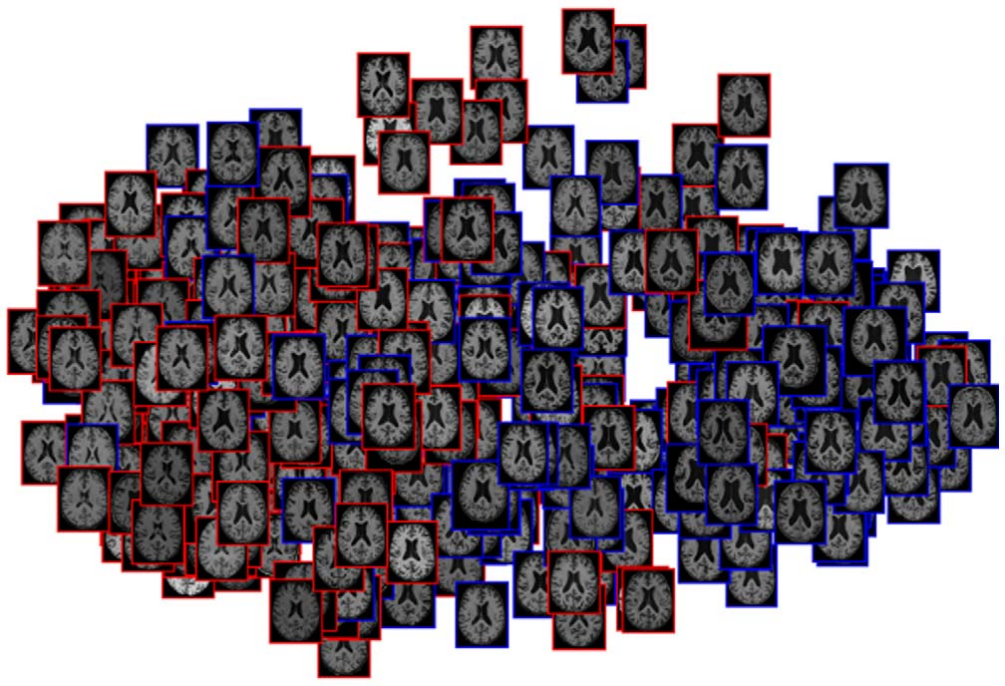
2D manifold embedding of a set of images acquired from healthy controls (red) and subjects with AD (blue).

In this machine learning approach, non-linear dimensionality reduction with Laplacian eigenmaps [48] is used to learn features to discriminate between different subject groups. Laplacian eigenmaps estimates the low-dimensional representation of a set of input images based on a similarity graph that is defined with pairwise image similarities [48]. The hypothesis is that such a low-dimensional representation captures the variability in the dataset in a more compact way than pairwise image similarities directly. We estimate pairwise image similarities from the intensity appearance in a region around hippocampus and amygdala since both structures are known to be affected by AD in an early stage. All images are aligned in a template space using a coarse non-rigid registration (10 mm B-spline control-point spacing, [49]). Such a coarse non-rigid alignment ensures that corresponding brain structures are aligned but still allows to measure subject-specific differences. After performing dimensionality reduction, the first 20 dimensions of the resulting manifold are used as features to perform classification with the different methods used. More details on the theory and application of this manifold learning approach can be found in [20], [50]. Figure 1 exemplarily shows a 2D embedding of a set of ADNI images acquired from healthy controls and subjects with AD. It can be seen that even two embedding dimensions give a relatively good separation between both groups. In our experiments we used a higher dimensional space allowing better discrimination.

### ROI-wise features for CTH and TBM

**Figure 2:**
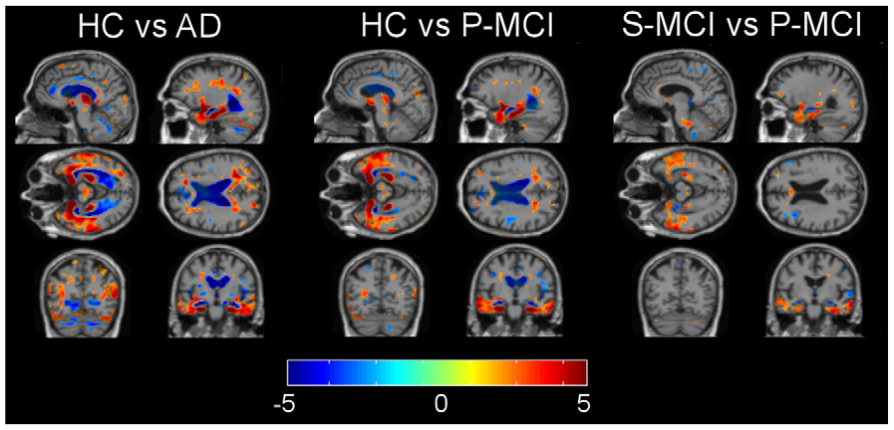
Results for voxelwise t-tests for statistically significant group differences with features extracted from TBM.

**Figure 3:**
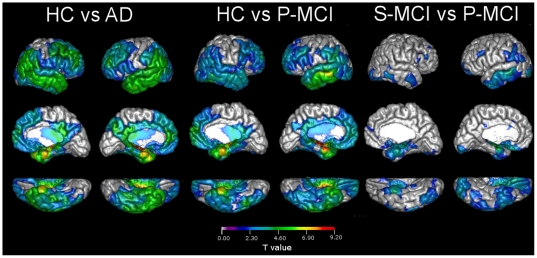
Results for t-tests for statistically significant group differences based on cortical thickness measurements.

Both CTH and TBM analyses produce local (point-wise) information, either on cortical thickness or the volume. Thus, the number of original features is enormous, and to make the classification more efficient and robust, the number of features has to be reduced. We evaluated both features in a statistical region of interest (ROI) defined as detailed in Appendix S2. Figures 2 and 3 show t-values for statistically significant differences between study groups for TBM and CTH respectively. A detailed description of the definition of these statistical ROIs is given in Appendix S2.

### Study design

Table 2 presents an overview on the features calculated for all 834 available ADNI baseline images. All feature values were corrected for age and gender using a linear regression model where control subjects were used as the training set, i.e., the normal, not disease-related, age and gender related differences in the classification features were removed. Feature selection was then carried out on the corrected feature sets using stepwise regression [51].

**Table 2:** Features used in the study.

Method	No of features	Description
Hippocampal volume (HV)	1	total volume of left and right hippopcampus
Cortical thickness	9 (HC vs AD)	average cortical thickness within a ROI defined based on group-level statistical analysis
(CTH)	7 (HC vs P-MCI)	
	8 (S-MCI vs P-MCI)	
Tensor-based morphometry (TBM)	84	average Jacobian of atrophic voxels within a ROI, weighted based on voxel-wise p-values
Manifold-based learning (MBL)	20	coordinates of a subject in a low-dimensional manifold space learned from pairwise image similarities

We used two subsets to perform classification:

- All 834 available baseline images described in the subjects section
- 509 baseline images used by Cuingnet et al. [29] and detailed in their publication.

The following sections describe the definition of the statistical ROIs and evaluation strategy used for the two datasets respectively.

#### Dataset I

In order to perform the study using cross-validation in the full dataset, it was divided into three equally sized parts. One part was used to perform the statistical tests for the CTH and TBM features, and the remaining two parts were used to evaluate the classification accuracy. This was repeated three times so that each part was once used to perform the statistical tests. Afterwards, the results of the three repetitions were averaged. The classification accuracy was evaluated using leave-N-out cross validation on those subjects not included in the statistical tests. Five percent of the evaluation subjects were regarded as the test set, and the remaining 95% of the subjects were used to train a classifier which was then applied to the test set. This was repeated table-1-caption100 times, each time selecting randomly the test set subjects. Finally, the results of the 100 repetitions were averaged. Consequently, in overall, the classification evaluation was performed using 300 (3
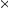
100) repetitions, and the results presented in this paper are the average values of all these classifications.

#### Dataset II

Statistical ROIs for CTH and TBM feature extraction were calculated from the 325 baseline images that are not part of dataset II. In order to allow direct comparison of classification accuracy with the work by Cuingnet et al. [29], separate training and testing sets for the different comparisons were defined using the exact sub-groups reported in their manuscript. Around 50% of all subjects are used to train the different types of classifiers and the reported results are based on classifying the remaining subjects.

### Classification methods

We used two different widely used methods to perform classification based on individual features and their combination:

#### Linear discriminant analysis (LDA)

Linear discriminant analysis (LDA) is a widely used technique to find a linear combination of features to best separate several classes [52]. In this work we used LDA as implemented in the *classify* function in Matlab with a multivariate normal density model with uninformative priors (p = 0.5).

#### Support vector machines (SVM)

Support vector machines use training data to find a separating hyperplane in the n-dimensional training space that best separates two subject groups [53]. Test subjects are then classified according to their position relative to the defined hyperplane in the n-dimensional feature space. We used the libSVM library to perform the analysis. The radial basis function kernel was selected based on the guidelines provided by the libSVM library (Software available 2.3.2011 at http://www.csie.ntu.edu.tw/cjlin/libsvm).

## Results

We used both classification methods to measure classification accuracy based on individual features as well as the combination of all features. The results for the comparisons HC vs AD, HC vs P-MCI and S-MCI vs P-MCI in the full ADNI database are presented in Tables 3, 4 and 5 respectively. Presented are classification accuracy (CCR), sensitivity (SEN) and specificity (SPE). Furthermore, the 95% confidence interval for the classification accuracy is estimated based on the multiple classification runs. Statistically significant improvements achieved when combining all features are marked with 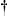 (p
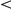
0.0001). To test for significance, unpaired t-tests were carried out between distribution estimates for the corresponding classification rates based on the multiple runs. All estimated distributions passed a normality test using a Kolmogorov-Smirnov test at 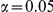.

**Table 3:** Classification results for HC vs AD.

Feature	LDA			SVM		
	CCR [95% CI]	SEN	SPE	CCR [95% CI]	SEN	SPE
MBL	85† [64 100]	87	83	85 [64 100]	87	83
HV	81† [57 100]	81	79	81† [57 100]	84	77
CTH	81† [64 100]	89	71	82† [57 100]	90	73
TBM	87† [71 100]	90	84	87 [71 100]	89	84
All	89 [71 100]	93	85	86 [71 100]	94	78

†means statistically significant different from the combined results with p
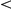
0.0001. CCR = Correct classification rate, SEN = Sensitivity, SPE = Specificity.

**Table 4:** Classification results for HC vs P-MCI.

Feature	LDA			SVM		
	CCR [95% CI]	SEN	SPE	CCR [95% CI]	SEN	SPE
MBL	78† [54 100]	81	75	77† [54 92]	84	69
HV	76† [54 92]	77	76	78 [54 92]	83	71
CTH	77† [54 100]	85	65	77 [54 100]	89	62
TBM	79† [62 100]	82	76	80† [62 100]	85	74
All	84 [62 100]	86	82	82 [62 100]	93	67

†means statistically significant different from the combined results with p
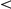
0.0001.

**Table 5:** Classification results for S-MCI vs P-MCI.

Feature	LDA			SVM		
	CCR [95% CI]	SEN	SPE	CCR [95% CI]	SEN	SPE
MBL	65† [36 86]	64	66	65† [43 86]	77	48
HV	65† [36 86]	63	67	62 [36 86]	83	33
CTH	56† [29 86]	63	45	59 [36 79]	96	03
TBM	64† [36 86]	65	62	64† [36 86]	77	44
All	68 [43 93]	67	69	60 [36 86]	92	14

†means statistically significant different from the combined results with p
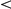
0.0001.

For direct comparison with work presented by Cuingnet et al. [29], we performed classification based on the training- and testing sets defined in their manuscript as described above. S-MCI and P-MCI groups are defined in the same way as in the original publication. Sensitivity and specificity values for the classification in all three clinical pairings are reported in Table 6. Following the clear advantage for LDA in the performance on the full dataset, we only report results with this classifier for dataset II.

**Table 6:** Classification results based on a subset of ADNI that was previously used for classification by Cuingnet et al. [29].

Feature	HC vs AD		HC vs P-MCI		S-MCI vs P-MCI	
	SEN	SPE	SEN	SPE	SEN	SPE
MBL	90	74	84	92	55	76
HV	80	69	75	76	63	70
CTH	85	75	86	59	72	35
TBM	93	76	90	84	63	59
All	94	76	94	89	69	54

## Discussion

In this study we assessed the automatic diagnostic capabilities of 4 structural MRI features (MBL, HC, CTH, TBM) separately and combined in 834 baseline images acquired in the ADNI study. When applied separately, TBM provided the overall best results, closely followed by MBL. Combining all features improved the results in all study experiments. Our results show how a combination of different MRI-based features can improve results based on only one measurement, resulting in a more powerful and stable classifier. The most significant improvement of the combination over the best individual feature can be observed for HC vs P-MCI with 5% units followed by 3 and 2% units for S-MCI vs P-MCI and HC vs AD, respectively. These improvements lead to 20, 12 and 9 subjects more being correctly classified respectively when using the combined feature set as compared to the best single feature for every comparison. Comparing two classification approaches based on LDA and SVMs resulted in a clear advantage of the former.

Several studies reported classification results using single MRI methods for the HC/AD classification (Table 7). Liu et al. [24] reported SEN/SPE of 92/90 in the classification of HC/AD subjects using regional cortical volumes in the AddNeuroMed dataset. McEvoy et al. [26] report a CCR of 89 on images from the ADNI database using features from cortical thickness and structural volumes. Vemuri et al. [54] present a SEN/SPE of 86/86 on 380 subjects using the STAND score. In our study the results obtained with single methods are lower (71–90) but almost identical when the methods were combined. It should be noted, however, that Liu and colleagues did not use cross-validation or separate training/testing sets when producing the results which could lead to overestimation of the results in a dataset outside the study cohort. Gerardin et al. [23] acquired a high SEN/SPE of 96/92 by using hippocampal shape analysis, but the number of subjects (25 HC, 23 AD) was quite low in order to produce results with good generalizability. Westman et al. [55] reported a CCR of 82 for HC vs AD classification and 73 for HC vs P-MCI classification by using various regional brain volumes. Our results are substantially more accurate, the group sizes are larger and clinical follow-up time is one year longer. Chupin et al. [21] reported SEN/SPE of 75/77 (hippocampal volume) and Querbes et al. [27] a CCR of 85 (cortical thickness), both lower than the results acquired with the combination of features or TBM features independently in our study.

**Table 7:** Classification results of healthy control (HC), mild cognitive impairment (MCI) and Alzheimer's disease subjects reported in the recent literature.

Study	N	Features	HC vs AD			HC vs P-MCI			S-MCI vs P-MCI		
			CCR	SEN	SPE	CCR	SEN	SPE	CCR	SEN	SPE
Liu et al. [24]	333	Cortical volumes	91	92	90	-	-	-	-	-	-
Gerardin et al. [23] *	70	Hippocampus shape	94	96	92	-	-	-	-	-	-
Chupin et al. [21] *	605	Hippocampus volume	76	75	77	-	-	-	64	60	65
Querbes et al. [27] *	382	Cortical thickness	85	-	-	-	-	-	73	75	68
Liu et al. [25]	312	Amygdala/caudate volumes	-	-	-	-	-	-	69	76	68
Davatzikos et al. [15] *	356	SPARE-AD index	-	-	-	-	-	-	56	95	38
Cuingnet et al. [29] *	509	Various	-	81	95	-	73	85	-	62	69
Hinrichs et al. [14] *	159	MRI & PET	81	-	-	60	92	14	-	-	-
Westman et al. [55]	351	Various volumes	82	-	-	73	-	-	-	-	-
McEvoy et al. [26] *	398	Cortical thickness/various volumes	89	83	93	-	-	-	-	-	-
Vemuri et al. [54]	380	STAND score	-	86	86	-	-	-	-	-	-

N = Number of study subjects,

* = ADNI dataset.

Varying results concerning AD prediction (S-MCI/P-MCI classification using baseline measurements) have been published (Table 7): Querbes et al. [27] reported a CCR of 73, Liu et al. [25] a SEN/SPE of 76/68, Chupin et al. [21] reported a SEN/SPE of 60/65 and Davatzikos et al. [15] SEN/SPE of 95/38. Our results with separate and combined baseline features lie in the range of these results (SEN/SPE 63/67, 64/66 and 67/69 when using HV, MBL and the combined features, respectively).

There can be several explanations for the variation in the reported results. A majority of the studies in this field have used different statistical methods and MRI feature extraction strategies on different datasets, which makes a comparison of the results complicated. Also the variation in the size of the study samples and the use (or ignoring) of cross-validation or separate training/testing sets are important factors, which both have crucial impact on the reliability and generalizability of the results. In Lötjönen et al. [36], we demonstrated that choosing from a population of 350 cases several times 2/3 for the training set and 1/3 for the test set and using hippocampus volume as a classification feature can lead to any classification accuracy between 53% and 77%. This observation is also confirmed by the high confidence intervals for the classification accuracies reported in Tables 3, 4 and 5. This shows that a fair comparison of methods based on the classification accuracy is difficult if not exactly the same data and classification approaches are used. Furthermore, since the ADNI study is still ongoing, several subjects labeled as S-MCI will progress in the future to the P-MCI group.

A recent study with a subset of ADNI subjects assessed the classification performance of several structural MRI methods in experiments comparable to our investigation [29]. Reported SEN/SPE lie in the ranges 59/81–81/95 (HC vs AD) and 70/73–73/85 (HC vs P-MCI). While most methods tested did not exceed the accuracy of a random classifier for the discrimination between S-MCI and P-MCI, the best results reported for this task were a SEN/SPE of 62/69 when using hippocampal volume. To allow a direct comparison of the results reported by Cuingnet et al. [29], we evaluated our features on the exact same training- and testing sets used in their paper. This direct comparison shows that our results compare favourably to other, established methods in neuroimaging. For HC vs AD classification, individual features in our study give more sensitive but less specific results than most methods in the previous publication. Combining all features gives an overall better classification accuracy than the majority of previously tested methods. Our results on the combined feature set furthermore outperform the majority of methods tested by Cuingnet et al. [29] when predicting MCI conversion as well as all methods for the classification between HC and P-MCI. A significant difference in classification accuracy can be observed between the full ADNI dataset and this smaller subset used for comparison with previous work. Reasons may include a strict separation into trainin- and testing sets which may result in less generalisability as well as the shorter follow-up period that was considered to define progression to AD.

Some studies have also combined different biomarkers (CSF, MRI, PET) with the idea of measuring different aspects of AD pathology and thus improve the classification accuracy. Hinrichs et al. [14] improved their HC/AD classification CCR by a few % units to 81 by combining MRI and PET. Eckerström et al. [16] studied the separation of a unified HC/S-MCI group from P-MCI group with CSF proteins and manual hippocampal volumes. They found CSF to be superior to MRI (SEN/SPE of 95/79 vs 86/66) while the combination performed best (SEN/SPE 90/91). However, it should be noted that the study sample in that particular study was small (a total of 68 subjects) and neither cross-validation or separate training/testing sets were used in order to ensure good generalizability of the results. In Kohannim et al. [17], the improvement from using multiple biomarkers was not significant and Davatzikos et al. [15] reported marginal improvements which, however, may be related to the fact that results with only one biomarker were not very good to begin with.

Considering solely the classification accuracies of the present study and those reported in literature, it seems questionable if the collection of several biomarkers is worth the effort and resource. A combination of different features extracted from a single MRI seems to provide results that are comparable or better than those obtained with other or multiple biomarkers. In a clinical point of view, this is interesting since it means that a single MRI scan provides not only aid to differential diagnostics of cognitive impairment, but also reliably describes a persons phase in the HC/AD continuum. MRI is also widely available, non-invasive and often useful in the differential diagnostics of memory problems thus making it a compelling option as the first biomarker that would be obtained from a patient with mild memory problems. However, a comprehensive differential diagnostics between AD and non-AD cognitive impairments will still require assessment of various different biomarkers. Also, it should be noted that the computational techniques used in this paper are not widely available in the clinical environment and thus limit their usage in the clinical work at present.

Strengths of the presented study are i) the use of multiple features extracted from one imaging modality, ii) large groups, iii) rigorous validation process of the results using cross-validation, and iv) results comparable or better than the ones published so far.

Our study has also some limitations that should be mentioned. The results are obtained from a single (although collected from multiple sites) cohort and should be also validated in other cohorts. A longer clinical follow-up time would be needed to see if the classification results of S-MCI/P-MCI experiment changed when more of the MCI subjects converted to AD. Furthermore, the ADNI study does not provide postmortem pathological confirmation of the clinical status. With this limitation, individual subjects might be wrongly categorized. Although a rigorous validation process was used, optimally we need to establish standardized cut-offs that would be well generalizable to other cohorts outside ADNI. That is, however, beyond the possibilities of this study and will require vast standardization and validation procedures. Also, the CTH pipeline had problems especially with severely atrophied brains or MRI scans with poor image quality. A more robust pipeline would be desirable in order to guarantee a more reliable feature extraction.

## Supporting Information

Appendix S1 The Alzheimer's Disease Neuroimaging Initiative.
(DOCX)

Appendix S2 ROI-wise features for CTH and TBM.
(DOCX)

## References

[1] Brookmeyer R, Johnson E, Ziegler-Graham K, Arrighi HM (2007). Forecasting the global burden of Alzheimer's disease.. Alzheimer's and Dementia.

[2] Cummings J, Doody R, Clark C (2007). Disease-modifying therapies for alzheimer disease: challenges to early intervention.. Neurology.

[3] Petersen R (2001). Practice parameter: early detection of dementia: mild cognitive impairment (an evidence-based review).. Neurology.

[4] Gauthier S, Reisberg B, Zaudig M, Petersen R, Ritchie K (2006). Mild cognitive impairment.. Lancet.

[5] Braak H, Braak E (1991). Neuropathological stageing of Alzheimer-related changes.. Acta Neuropathologica.

[6] Wenk G (2003). Neuropathologic changes in alzheimer's disease.. Journal of Clinical Psychiatry.

[7] Hampel H, Brger K, Teipel SJ, Bokde AL, Zetterberg H (2008). Core candidate neurochemical and imaging biomarkers of alzheimer's disease.. Alzheimer's and Dementia.

[8] Hyman BT, Marzloff K, Arriagada PV (1993). The lack of accumulation of senile plaques or amyloid burden in Alzheimer's disease suggests a dynamic balance between amyloid deposition and resolution.. J Neuropathol Exp Neurol.

[9] Gmez-Isla T, Hollister R, West H, Mui S, Growdon J (1997). Neuronal loss correlates with but exceeds neurofibrillary tangles in alzheimer's disease.. Ann Neurol.

[10] Ingelsson M, Fukumoto H, Newell KL, Growdon JH, Hedley-Whyte ET (2004). Early abeta accumulation and progressive synaptic loss, gliosis, and tangle formation in ad brain.. Neurology.

[11] Jack C, Knopman D, Jagust W, Shaw L, Aisen P (2010). Hypothetical model of dynamic biomarkers of the alzheimer's pathological cascade.. Lancet Neurology.

[12] Hampel H, Frank R, Broich K, Teipel S, Katz J, abd Hardy RG (2010). Biomarkers for alzheimer's disease: academic, industry and regulatory perspectives.. NatRevDrug Discov.

[13] Fan Y, Resnick SM, Wu X, Davatzikos C (2008). Structural and functional biomarkers of prodromal alzheimer's disease: A high-dimensional pattern classification study.. NeuroImage.

[14] Hinrichs C, Singh V, Xu G, Johnson S (2009). Mkl for robust multi-modality ad classification.. MICCAI (II).

[15] Davatzikos C, Bhatt P, Shaw LM, Batmanghelich KN, Trojanowski JQ (2010). Prediction of MCI to AD conversion, via MRI, CSF biomarkers, and pattern classification..

[16] Eckerstrom C, Andreasson U, Olsson E, Rolstad S, Blennow K (2010). Combination of hippocampal volume and cerebrospinal uid biomarkers improves predictive value in mild cognitive impairment.. Dement Geriatr Cogn Disord.

[17] Kohannim O, Hua X, Hibar DP, Lee S, Chou YY (2010). Boosting power for clinical trials using classifiers based on multiple biomarkers.. Neurobiology of Aging.

[18] Landau S, Harvey D, Madison C, Reiman E, Foster N (2010). Comparing predictors of conversion and decline in mild cognitive impairment.. Neurology.

[19] Walhovd KB, Fjell AM, Brewer J, McEvoy LK, Fennema-Notestine C (2010). Combining MR Imaging, Positron-Emission Tomography, and CSF Biomarkers in the Diagnosis and Prognosis of Alzheimer Disease.. American Journal of Neuroradiology.

[20] Wolz R, Aljabar P, Hajnal JV, Lotjonen J, Rueckert D (2011). Manifold learning combining imaging with non-imaging information.. IEEE International Symposium on Biomedical Imaging.

[21] Chupin M, Hammers A, Liu R, Colliot O, Burdett J (2009). Automatic segmentation of the hippocampus and the amygdala driven by hybrid constraints: Method and validation.. NeuroImage.

[22] Devanand DP, Pradhaban G, Liu X, Khandji A, De Santi S (2007). Hippocampal and entorhinal atrophy in mild cognitive impairment - Prediction of Alzheimer disease.. Neurology.

[23] Gerardin E, Chetelat G, Chupin M, Cuingnet R, Desgranges B (2009). Multidimensional classification of hippocampal shape features discriminates Alzheimer's disease and mild cognitive impairment from normal aging.. NeuroImage.

[24] Liu Y, Paajanen T, Zhang Y, Westman E, Wahlund LO (2009). Combination analysis of neuropsychological tests and structural MRI measures in differentiating AD, MCI and control groups–The AddNeuroMed study..

[25] Liu Y, Paajanen T, Zhang Y, Westman E, Wahlund LO (2010). Analysis of regional MRI volumes and thicknesses as predictors of conversion from mild cognitive impairment to Alzheimer's disease.. Neurobiology of Aging.

[26] McEvoy L, Fennema-Notestine C, JC R, Hagler D, Holland D (2009). Alzheimer disease: quantitative structural neuroimaging for detection and prediction of clinical and structural changes in mild cognitive impairment.. Radiology.

[27] Querbes O, Aubry F, Pariente J, Lotterie JA, Dmonet JF (2009). Early diagnosis of alzheimer's disease using cortical thickness: impact of cognitive reserve.. Brain.

[28] Mueller SG, Weiner MW, Thal LJ, Petersen RC, Jack C (2005). The Alzheimer's Disease Neuroimaging Initiative.. Neuroimaging Clinics of North America.

[29] Cuingnet R, Gerardin E, Tessieras J, Auzias G, Lehricy S (2010). Automatic classification of patients with Alzheimer's disease from structural MRI: A comparison of ten methods using the ADNI database..

[30] Petersen RC, Aisen PS, Beckett LA, Donohue MC, Gamst AC (2010). Alzheimer's Disease Neuroimaging Initiative (ADNI): clinical characterization.. Neurology.

[31] Folstein MF, Folstein SE, McHugh PR (1975). Mini-mental state: A practical method for grading the cognitive state of patients for the clinician.. Journal of Psychiatric Research.

[32] Morris J (1993). The Clinical Dementia Rating (CDR): current version and scoring rules.. Neurology.

[33] McKhann G, Drachman D, Folstein M, Katzman R, Price D (1984). Clinical diagnosis of alzheimer's disease: report of the nincds-adrda work group under the auspices of department of health and human services task force on alzheimer's disease.. Neurology.

[34] Jack CR, Bernstein MA, Fox NC, Thompson P, Alexander G (2008). The Alzheimer's disease neuroimaging initiative (ADNI): MRI methods.. Journal of Magnetic Resonance Imaging.

[35] Lotjonen JM, Wolz R, Koikkalainen JR, Thurfjell L, Waldemar G (2010). Fast and robust multi-atlas segmentation of brain magnetic resonance images.. NeuroImage.

[36] Lotjonen J, Wolz R, Koikkalainen J, Julkunen V, Thurfjell L (2011). Fast and robust extraction of hippocampus from mr images for diagnostics of alzheimer's disease.. NeuroImage.

[37] Van Leemput K, Maes F, Vandermeulen D, Suetens P (1999). Automated model-based tissue classification of MR images of the brain.. IEEE Transactions on Medical Imaging.

[38] Lerch J, Evans A (2005). Cortical thickness analysis examined through power analysis and a population simulation.. NeuroImage.

[39] Mazziotta J, Toga A, Evans A, Fox P, Lancaster J (2001). A probabilistic atlas and reference system for the human brain: International Consortium for Brain Mapping (ICBM).. Philos Trans R Soc Lond B Biol Sci.

[40] Sled JG, Zijdenbos AP, Evans AC (1998). A nonparametric method for automatic correction of intensity nonuniformity in MRI data.. IEEE Transactions on Medical Imaging.

[41] Smith SM (2002). Fast robust automated brain extraction.. Hum Brain Mapp.

[42] Zijdenbos AP, Forghani R, Evans AC, III WMW, Colchester ACF, Delp SL (1998). Automatic quantification of MS lesions in 3D MRI brain data sets: Validation of INSECT.. MICCAI.

[43] Tohka J, Zijdenbos A, Evans A (2004). Fast and robust parameter estimation for statistical partial volume models in brain mri.. NeuroImage.

[44] Kim JS, Singh V, Lee JK, Lerch J, Ad-Dab'bagh Y (2005). Automated 3-d extraction and evaluation of the inner and outer cortical surfaces using a laplacian map and partial volume effect classification.. NeuroImage.

[45] Chung M, Taylor J (2004). Diffusion smoothing on brain surface via finite element method.. ISBI.

[46] Brun CC, Lepor N, Pennec X, Lee AD, Barysheva M (2009). Mapping the regional inuence of genetics on brain structure variability – a tensor-based morphometry study.. NeuroImage.

[47] Koikkalainen J, Lotjonen J, Thurfjell L, Rueckert D, Waldemar G (2011). Multi-template tensor-based morphometry: Application to analysis of alzheimer's disease.. NeuroImage.

[48] Belkin M, Niyogi P (2003). Laplacian eigenmaps for dimensionality reduction and data representation.. Neural Computation.

[49] Rueckert D, Sonoda LI, Hayes C, Hill DLG, Leach MO (1999). Nonrigid registration using free-form deformations: Application to breast MR images.. IEEE Transactions on Medical Imaging.

[50] Wolz R, Heckemann RA, Aljabar P, Hajnal JV, Hammers A (2010). Measurement of hippocampal atrophy using 4D graph-cut segmentation: Application to ADNI.. NeuroImage.

[51] Draper NR, Smith H (1998). Applied Regression Analysis (Wiley Series in Probability and Statistics).. Wiley.

[52] Krzanowski WJ (1988). Principles of Multivariate Analysis: A User's Perspective.

[53] Cortes C, Vapnik V (1995). Support-vector networks.. Machine Learning.

[54] Vemuri P, Gunter JL, Senjem ML, Whitwell JL, Kantarci K (2008). Alzheimer's disease diagnosis in individual subjects using structural mr images: Validation studies.. NeuroImage.

[55] Westman E, Simmons A, Zhang Y, Muehlboeck JS, Tunnard C (2011). Multivariate analysis of mri data for alzheimer's disease, mild cognitive impairment and healthy controls.. NeuroImage.

