# Supplementary material for: Multi-Method Analysis of MRI Images in Early Diagnostics of Alzheimer's Disease
Source: PLoS One. 2011 Oct 13;6(10):e25446. doi: 10.1371/journal.pone.0025446 (PMC3192759; doi:10.1371/journal.pone.0025446)
Supplement: Appendix S1 — The Alzheimer's Disease Neuroimaging Initiative. (DOCX) [file pone.0025446.s001.docx]

**The Alzheimer's Disease Neuroimaging Initiative**

The Alzheimer's Disease Neuroimaging Initiative (ADNI) was launched in 2003 by the National Institute on Aging (NIA), the National Institute of Biomedical Imaging and Bioengineering (NIBIB), the Food and Drug Administration (FDA), private pharmaceutical companies and non-profit organizations, as a 60 million, 5-year public-private partnership. The primary goal of ADNI has been to test whether serial MRI, positron emission tomography (PET), other biological markers, and clinical and neuropsychological assessment can be combined to measure the progression of MCI and AD. Determination of sensitive and specific markers of very early AD progression is intended to aid researchers and clinicians to develop new treatments and monitor their effectiveness, as well as lessen the time and cost of clinical trials.

The Principle Investigator of this initiative is Michael W. Weiner, M.D., VA Medical Center and University of California - San Francisco. ADNI is the result of efforts of many co-investigators from a broad range of academic institutions and private corporations, and subjects have been recruited from over 50 sites across the U.S. and Canada. The initial goal of ADNI was to recruit 800 adults, ages 55 to 90, to participate in the research -- approximately 200 cognitively normal older individuals to be followed for 3 years, 400 people with MCI to be followed for 3 years, and 200 people with early AD to be followed for 2 years. For up-to-date information see www.adni-info.org.
