# Supplementary material for: Multi-Method Analysis of MRI Images in Early Diagnostics of Alzheimer's Disease
Source: PLoS One. 2011 Oct 13;6(10):e25446. doi: 10.1371/journal.pone.0025446 (PMC3192759; doi:10.1371/journal.pone.0025446)
Supplement: Appendix S2 — ROI-wise features for CTH and TBM. (DOCX) [file pone.0025446.s002.docx]

### ROI-wise features for CTH and TBM

Both CTH and TBM analyses produce local (point-wise) information, either on cortical thickness or the volume. Thus, the number of original features is enormous, and to make the classification more efficient, the number of features has to be reduced. A ROI-based approach utilizing an anatomical atlas of 83 structures [1,2] was selected. Both the CTH nodes and the TBM voxels were mapped to a single structure, and the CTH and the Jacobian values were averaged within each ROI. However, as shown in [3], the classification results can be improved by focusing on the statistically significant nodes or voxels. Therefore, statistically significant locations were determined by applying t-test on a separate training set. The feature value of the *r*th ROI for the *i*th subject was then computed from

$F\left( i,r \right)= \frac{\sum_{\vec{p}\in r\cap T\left( \vec{p} \right)>0} W\left( \vec{p} \right)\cdot f_{i}\left( \vec{p} \right)-\sum_{\vec{p}\in r\cap T\left( \vec{p} \right)<0} W\left( \vec{p} \right)\cdot f_{i}\left( \vec{p} \right)}{\sum_{\vec{p}\in r} W\left( \vec{p} \right)}$,

where $T(\vec{p})$ is the t-value of the t-test in the voxel/node $\vec{p}$ and $W\left( \vec{p} \right)$ is a weighting function defined as

$W\left( \vec{p} \right)=\frac{\log\left( p_{max} \right)-log(P\left( \vec{p} \right))}{\log\left( p_{max} \right)-\log\left( p_{min} \right)}$,

where $P(\vec{p})$ is the p-value, $p_{max}=0.05$ and $p_{min}=0.000001$ are user-defined parameters, and the p-values are constrained to the interval defined by $p_{min}$ and $p_{max}$. The computation of the feature values was done separately for dilating ($T\left( \vec{p} \right)>0$) and shrinking ($T\left( \vec{p} \right)<0$) voxels/nodes so that they would not cancel each other within a ROI. The weighting was used to focus the computations on the voxels that have statistically significant group level differences. It gives a voxel a larger weight the smaller the p-value is, and no weight is given to the statistically non-significant voxels. The threshold $p_{min}$ was used to avoid situations where just a few highly significant voxels would have a too large impact on a feature value. In TBM, the final features for the classification were obtained by averaging the values $F(i,r)$ of the 30 templates, i.e., ROI-wise average feature values were used. To avoid unnecessary complexity in the classification, a subset of the 83 ROIs were selected for both CTH and TBM. Tables 1 and 2 give an overview on the anatomical regions that were used to analyse TBM and CTH features respectively. All structures were selected both in the left and the right hemisphere. A detailed anatomical definition of all structures can be found in [1] and [2] (www.brain-development.org).

1. Hammers A, Allom R, Koepp MJ, Free SL, Myers R, et al. (2003) Three-dimensional maximum

probability atlas of the human brain, with particular reference to the temporal lobe. Human Brain

Mapping 19: 224{247.

2. Gousias IS, Rueckert D, Heckemann RA, Dyet LE, Boardman JP, et al. (2008) Automatic

segmentation of brain mris of 2-year-olds into 83 regions of interest. NeuroImage 40: 672 - 684.

3. Koikkalainen J, Lotjonen J, Thurfjell L, Rueckert D, Waldemar G, et al. (2011) Multi-template

tensor-based morphometry: Application to analysis of alzheimer's disease. NeuroImage 56: 1134 –

1144.

Table 1: ROIs used in TBM analysis

| Hippocampus |
| --- |
| Amygdala |
| Anterior temporal lobe, medial part |
| Anterior temporal lobe, lateral part |
| Gyri parahippocampalis et ambiens |
| Superior temporal gyrus, posterior part |
| Medial and inferior temporal gyri |
| Insula |
| Posterior temporal lobe |
| Lateral ventricle, frontal horn, central part and occipital horn |
| Lateral ventricle, temporal horn |

Table 2: ROIs used in CTH analysis

| Anterior temporal lobe, medial part |
| --- |
| Anterior temporal lobe, lateral part |
| Gyri parahippocampalis et ambiens |
| Superior temporal gyrus, posterior part |
| Medial and inferior temporal gyri |
| Lateral occipitotemporal gyrus, gyrus fusiformis |
| Insula |
| Occipital lobe |
| Cingulate gyrus, anterior part |
| Cingulate gyrus, posterior part |
| Frontal lobe , becomes middle frontal gyrus after subdivision of frontal lobe |
| Posterior temporal lobe |
| Parietal lobe |
| Precentral gyrus |
| Straight gyrus, gyrus rectus |
| Anterior orbital gyrus |
| Inferior frontal gyrus |
| Superior frontal gyrus |
| Postcentral gyrus |
| Superior parietal gyrus |
| Lingual gyrus |
| Cuneus |
| Medial orbital gyrus |
| Lateral orbital gyrus |
| Posterior orbital gyrus |
| Subgenual frontal cortex |
| Pre-subgenual frontal cortex |
| Superior temporal gyrus, anterior part |
